# In-situ synthesis of phthalocyanines on electrospun TiO_2_ nanofiber by solvothermal process for photocatalytic degradation of methylene blue

**DOI:** 10.3906/kim-2108-14

**Published:** 2021-10-22

**Authors:** Selin GÜMRÜKÇÜ, Mukaddes ÖZÇEŞMECİ, Esma SEZER, Belkıs USTAMEHMETOĞLU, Esin HAMURYUDAN

**Affiliations:** Department of Chemistry, Faculty of Science and Letters, İstanbul Technical University, İstanbul, Turkey

**Keywords:** Phthalocyanine, nanofiber, electrospinning, photodegradation, photocatalytic

## Abstract

Titanium dioxide/phthalocyanine (TiO_2_/Pc), TiO_2_/fluor containing phthalocyanine (TiO_2_/FPc), and TiO_2_/fluor containing cobalt phthalocyanine (TiO_2_/FCoPc) had been successfully fabricated by a simple combination of phthalocyanines obtained by in-situ synthesis on the surface of TiO_2_ nanofibers prepared by electrospinning. Scanning electron microscopy micrographs and X-ray diffraction analysis indicated that the phthalocyanines uniformly immobilized on the surface of TiO_2_ nanofibers. Photocatalytic activity of TiO_2_, TiO_2_/Pc, TiO_2_/FPc, TiO_2_/FCoPc nanofibers for methylene blue in water was comparatively investigated firstly by ultraviolet-visible absorption measurements with time, and kinetic parameters were calculated. Results indicated that the obtained TiO_2_/Pc, TiO_2_/ FPc and TiO_2_/FCoPc exhibited high photocatalytic activity for the degradation of methylene blue and TiO_2_/FCoPc was found the best. It showed similar or higher activities than related studies and can be suggested as a promising candidate for environmental and energy applications.

## 1. Introduction

Synthetic organic dyes are widely used in chemical, petrochemical, food processing or textile industries, many of which are discharged to the environment by wastewater, causing many ecological problems [1–3]. In recent years, the removal of these dyes from wastewater has become a major and urgent need for a clean and comfortable environment. Several methods including chemical oxidation [4], adsorption [5], electrochemical treatment [6], microbiological degradation [7], and photocatalytic degradation [8] have been developed to resolve these problems. Among these methods, photocatalysis is one of the most attractive methods in this field because of its low cost and the formation of non-toxic by-products [9,10].

Titanium dioxide (TiO_2_) is one of the most important semiconductor materials and photocatalyst because of its low cost, easy availability, high chemical stability, anti-corrosive properties, and non-toxicity [11, 12].

Phthalocyanines (Pcs) are also known as attractive alternatives for photocatalytic decomposition based on the visible light of organic dyes. They have two absorptions in the UV region of 300–400 nm (B band) and 600–700 nm region (Q band) resulting from π-π* transitions. In addition, besides this property, metallophthalocyanines possess excellent resistance to chemical degradation and good photosensitivity [13–17]. The synthetic flexibility of phthalocyanines offers great possibilities to change the desired properties. For example, fluoro-substituted metallophthalocyanines have been reported to be efficient catalysts for many applications [18–22]. Electron withdrawing fluorine substituents decrease the electron density of the macrocyclic ring and increase the redox potential, catalytic activity, and stability [18–28].

Pc nanostructures were successfully grown on the TiO_2_ nanofibers substrates by in-situ one-pot synthesis with the advantage of saving time and simplicity.

Electrospinning is a way of obtaining nanofibers from various polymer solutions and melting them by applying electrical forces [29–31]. There are remarkable studies on the preparation of electrospun nanofibers to suit or enable various applications by changing the solution and processing parameters during production [32, 33]. Nanofibers are of considerable interest in a wide range of applications nowadays, including catalyst supports, drug delivery systems, sensors/ biosensors, and photocatalysts, due to the multiple benefits they provide [34–36]. This approach makes it possible to produce nanofibers by combining many objects with a mixture of different polymers or by making modifications to the surfaces of the resulting fibers [37, 38].

Because of the above advantages, combining the photoresponsive property of both TiO_2_ and phthalocyanines to prepare a photocatalyst is important in the degradation of organic dyes. In this study, methylene blue (MB) was studied because MB is a common industrial organic dye and overdose of MB dye may cause harmful effects on human health, such as accelerated heart rhythm, vomiting, tissue necrosis [3, 39, 40]. In these considerations, here, we attempted to design photocatalysts that can be efficient in the photocatalytic degradation of methylene blue (MB) under ultraviolet (UV) irradiation. Also, it aims to evaluate the photocatalytic efficiency of both the central metal cation as cobalt and the fluorinated peripheral groups. For these purposes, in this study, pure TiO_2_, TiO_2_/phthalocyanine (TiO_2_/Pc), TiO_2_/2,9(10),16(17),23(24)-tetrakis{[2′,3′,5′,6′-tetrafluoro-4′-(octafluoropentoxy)benzyloxy]phthalocyanine (TiO_2_/FPc) and TiO_2_/2,9(10),16(17),23(24)-tetrakis{[2′,3′,5′,6′-tetrafluoro-4′-(octafluoropentoxy) benzyloxy]phthalocyaninato} cobalt(II) (TiO_2_/FCoPc) photocatalysts based on TiO_2_ nanofibers were prepared by combining the electrospinning and solvothermal techniques. These photocatalysts were characterized by using X-ray diffraction (XRD), scanning electron microscopy (SEM), energy dispersive X-ray (EDX), and UV–Visible absorption spectra. Their photocatalytic properties were investigated by UV-Visible absorption spectrophotometric measurements, and kinetic parameters were obtained. Recycling properties of the photocatalytic degradation of MB using TiO_2_/FCoPc as the optimum photocatalyst was tested in order to gain information about multiple uses and stability of this material.

## 2. Experimental

### 2.1. Materials

Polyvinylpyrrolidone (PVP, M_w_ = 1.300.000) powder, phthalonitrile, ethanol, acetic acid, titanium(IV) butoxide (TBT), ethylene glycol, methylene blue (MB) were obtained from Sigma Aldrich. They were all used without any purification. Distilled water was supplied from Merck Millipore Milli-Q ultrapure water system (Merck Millipore, Molsheim, France). Distilled water was used throughout all experiments. 4-[2′,3′,5′,6′-tetrafluoro-4′-(octafluoropentoxy)benzyloxy] phthalonitrile was synthesized and purified as our previous report [25].

### 2.2. Preparation of photocatalysts

#### 2.2.1. Preparation of TiO_2_ nanofibers

**Figure 1:**
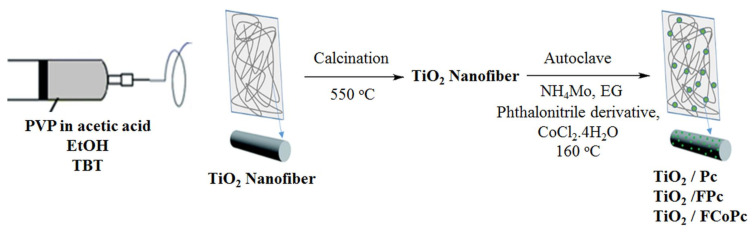
The schematic procedure of preparation of the TiO_2_/Pc, TiO_2_/FPc, and TiO_2_/FCoPc nanofibers.

In a capped bottle, 2 g PVP powder was dissolved in acetic acid (5 mL) and absolute ethanol (9 mL) mixture under vigorous stirring for 2 h. Then, TBT (2 g) was added to this homogeneous solution followed by stirring for 2 more hours to make a precursor solution. In order to have TiO_2_ nanofibers, a flat, aluminum foil-covered plate was located at a fixed distance of 15 cm from the needle tip. 12 kV (Gamma High Voltage Research) voltage was supplied, and electrospinning was carried out at room temperature. The feeding rate of the PVP solution was controlled by a digitally controlled syringe pump (New Era, NE-300), which was adjusted to a volume flow ratio of 1 mL/h. With the applied voltage, the solvent was evaporated and charged polymers were deposited on the Al foil collector in the form of nanofibers. TiO_2_ nanofibers were made by the calcination of obtained nanofibers at 550 °C for 2 h (Figure 1).

#### 2.2.2. Fabrication of TiO_2_/Pc and TiO_2_/FPc nanofibers

A mixture of TiO_2_ nanofibers (15 mg), ammonium molybdate (1 mg), phthalonitrile derivatives (phthalonitrile (12.8 mg) for TiO_2_/Pc or 4-[2′,3′,5′,6′-tetrafluoro-4′-(octafluoropentoxy)benzyloxy]phthalonitrile (53.6 mg) for TiO_2_/FPc and ethylene glycol (40 mL) were put into a teflon-lined stainless autoclave with the capacity of 50 mL. The autoclave was closed, and the reaction mixture was reacted at 160 °C for 20 h. The reaction system was closed, and the mixture was allowed to reach room temperature. The obtained samples were washed with distilled water under ultrasound (at three times) and ethyl alcohol to remove any ionic residue. Finally, the products were dried in vacuo (Figure 1).

#### 2.2.3. Fabrication of TiO_2_/FCoPc nanofibers

The autoclave was charged with 4-[2′,3′,5′,6′-tetrafluoro-4′-(octafluoropentoxy)benzyloxy]phthalonitrile (53.6 mg), CoCl_2_.4H_2_O (5 mg), ammonium molybdate (1 mg), TiO_2_ nanofibers (15 mg) and ethylene glycol (40 mL). This mixture was stirred at 160 °C for 24 h, then the reaction system was cooled down to room temperature. The product was washed successively with distilled water and ethanol, then, dried at 50 °C for 10 h (Figure 1).

### 2.3. Characterization

Surface morphologies of the fabricated nanofibers were investigated using a scanning electron microscope (SEM; QUANTA 400 F) after sputter coating with ultra-thin gold film. Energy dispersive X-ray (EDX) spectroscopy was used to analyze the composition of samples. Crystal structure analysis was carried out using X-ray diffraction (XRD) (Rigaku D/Max-IIIC diffractometer) with Cu-Kα line of 1.54 Å radiation and 2θ range of 10–90°. UV–Visible absorption spectra were recorded on a Perkin Elmer Lambda 45 UV-Visible spectrophotometer.

### 2.4. Photocatalytic degradation test

The photodegradation studies of MB solution were examined under the UV lamp by using synthesized nanofibers.

Photocatalytic degradation of MB for different nanofibers was investigated

- in the dark with the presence of photocatalysts,
- in the UV-light irradiation with the presence of the photocatalysts.

UV-A light (= 365 nm, UV-A 320 nm to 400 nm) was used in all the experiments [41]. Photocatalytic degradation of the MB was performed in a petri dish containing 100 mL dye solution with 10 mg of the photocatalyst sample. In order to figure out the photocatalysis activity of the samples, 5 ppm MB aqueous solution was prepared. The solution was stirred in dark for 30 min to realize adsorption-desorption equilibrium between the photocatalyst sample and the organic molecules. Changes in the concentrations of dyes were measured using UV-Vis spectrometer. The reaction was carried out at room temperature for 2 h. Samples of MB dye solution at different time intervals were analyzed during this time.

## 3. Results and discussion

**Figure 2:**
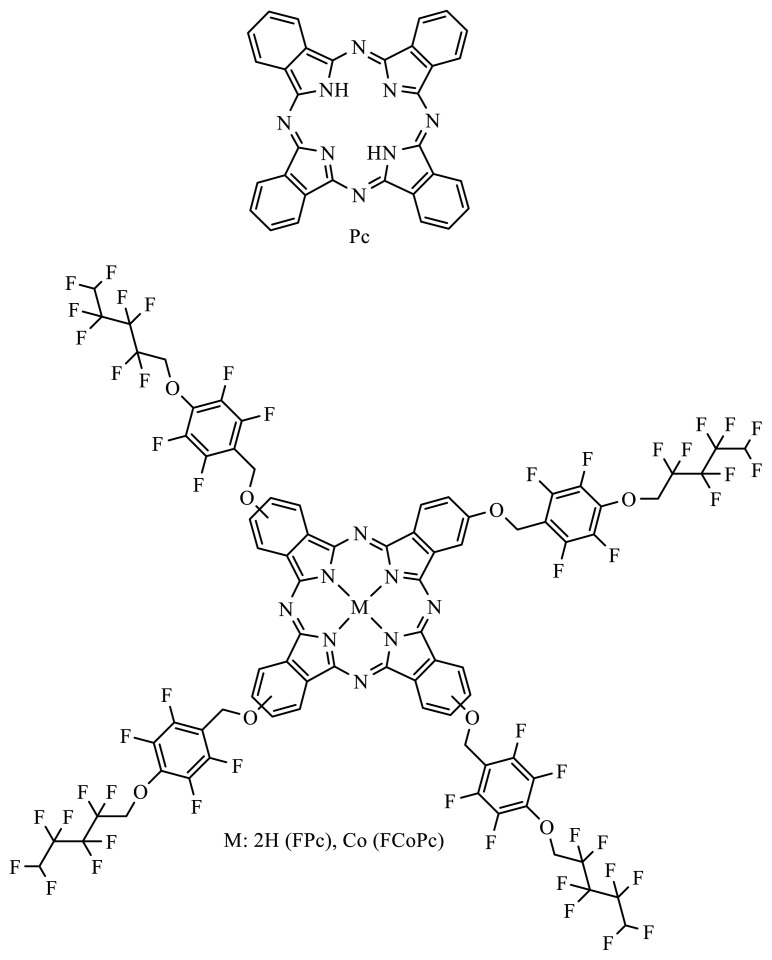
The structures of Pc, FPc, and FCoPc complexes.

In this work, pure TiO_2_, TiO_2_/Pc, TiO_2_/FPc, and TiO_2_/FCoPc nanofibers (Figure 2) were synthesized by combining the electrospinning and solvothermal techniques, and resulting nanofibers were characterized by SEM and XRD measurements. Detailed investigation of photocatalytic degradation of MB by using these nanofibers was also performed by kinetic measurements.

**Figure 3:**
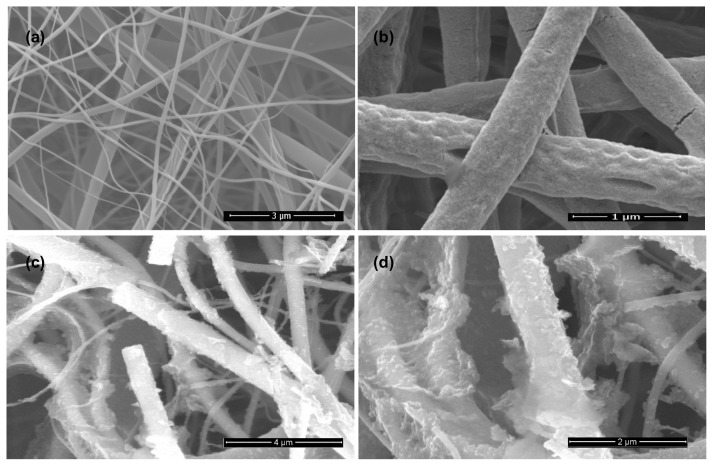
SEM images of **(a)** TiO_2_, **(b)** TiO_2_/Pc, **(c)** TiO_2_/FPc, and **(d)** TiO_2_/FCoPc nanofibers.

The morphological properties of these nanofibers were investigated by SEM (Figure 3). Figure 3a shows SEM images of the electrospun TiO_2_ nanofibers before solvothermal treatment. SEM images of calcinated TiO_2_ nanofibers at 550°C suggested smooth surface nanofibers with diameters in the range of 250–350 nm. The fabricated sample appeared as a non-woven nanofiber morphology after solvothermal treatment. A comparison between the SEM images of TiO_2_/Pc, TiO_2_/FPc and TiO_2_/FCoPc nanofibers in Figures 3b–3d, respectively with TiO_2_ nanofibers clearly indicating that different types of Pc nanostructures were grown on the surface of TiO_2_ nanofibers and gained beneficial properties such as large surface-to-volume ratio morphology. The morphology of FPc and FCoPc nanostructures grown on TiO_2_ nanofibers changed from the nanowires to nanoflowers in Figures 3c and 3d, compatible with results from the literature [42].

It is clearly seen that different types of Pc nanostructures grow on the surface of TiO_2_ nanofibers when different precursors are used according to the SEM images of TiO_2_/Pc, TiO_2_/FPc, and TiO_2_/FCoPc nanofibers in Figures 3b–3d. The average diameters of the TiO_2_, TiO_2_/Pc, TiO_2_/FPc, and TiO_2_/FCoPc nanofibers were calculated from the SEM images and found approximately as 0.45, 0.5, 0.7, and 0.8 μm, respectively. It shows that Pc nanostructures did not grow on the surface of TiO_2_ nanofibers when phthalonitrile compound was used as a precursor in the preparation of TiO_2_/Pc photocatalyst. It was clearly seen that when using 4-[2′,3′,5′,6′-tetrafluoro-4′-(octafluoropentoxy)benzyloxy]phthalonitrile (for TiO_2_/FPc) FPc nanostructures grow on the surface of TiO_2_ nanofibers. In addition, 4-[2′,3′,5′,6′-tetrafluoro-4′-(octafluoropentoxy) benzyloxy]phthalonitrile used in the preparation of the TiO_2_/FCoPc photocatalyst were reacted with CoCl_2_.4H_2_O to synthesize FCoPc molecules in situ. FCoPc molecules were collected as nanoflowers on the surface of TiO_2_ nanofibers. In the case of TiO_2_/FPc and TiO_2_/FCoPc nanofibers, 4-[2′,3′,5′,6′-tetrafluoro-4′-(octafluoropentoxy)benzyloxy]phthalonitrile was homogeneously dispersed in TiO_2_ nanofibers by the interaction of hydrogen bonds formed between the fluorinated groups of the phthalonitrile derivative and the surface hydroxyl groups of TiO_2_ nanofibers. Since the presence of Co in addition to fluorinated groups also enhances the interaction, the TiO_2_/FCoPc nanofiber has the largest diameter, hence the largest surface area, and increased interaction with MB is expected.

According to EDX analysis results of TiO_2_, TiO_2_/Pc, TiO_2_/FPc, and TiO_2_/FCoPc nanofibers, the weight percentage (Wt %) of each element was shown in Table 1. Ti and O elements were reported to exist in pure TiO_2_ electrospun nanofibers; on the other hand, Ti, N, F, and Co occurred in TiO_2_/Pc, TiO_2_/FPc, and TiO_2_/FPc nanofibers, respectively.

**Figure 4:**
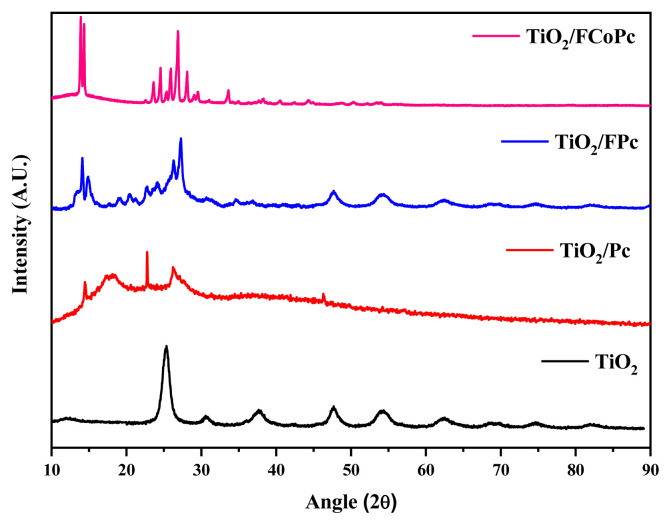
XRD patterns of TiO_2_, TiO_2_/Pc, TiO_2_/FPc and TiO_2_/ FCoPc nanofibers.

The crystal structures of TiO_2_, TiO_2_/Pc, TiO_2_/FPc, and TiO_2_/FCoPc nanofiber photocatalyst were characterized via X-ray diffraction spectrometer (XRD) (Figure 4). It was observed that the developed pure TiO_2_ nanofiber was a combination of anatase and brookite rather than a fully anatase structure. Anatase peaks at 25.3° (101), 37.9° (004), 47.7° (200), and 54.7° (002) and a minor brookite phase at 30.8° (121) can be seen [43]. As suggested in the literature, the presence of broad peaks around 14.8° (200), 21.1° to 30.4° (100), and at 42° to 46° (100) [44, 45] for TiO_2_/FCoPc and broad peaks around 15.7° to 30.2° (100) [46] for TiO_2_/FPc support to the formation of different Pc’s on TiO_2_ nanofiber.

**Figure 5:**
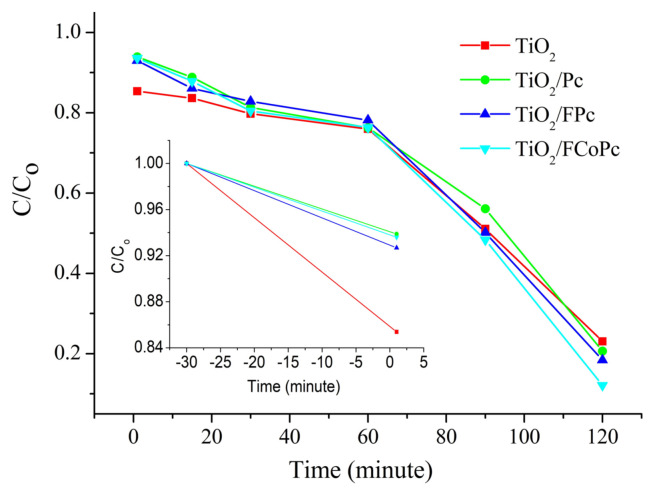
C/C_o_–time graphs for the photocatalytic degradation of MB for TiO_2_, TiO_2_/Pc, TiO_2_/FPc and TiO_2_/FCoPc nanofibers.

The performances of pure TiO_2_, TiO_2_/Pc, TiO_2_/FPc, and TiO_2_/FCoPc nanofiber photocatalysts were also evaluated on the degradation of MB that is a typical dye pollutant in industrial wastewater. Photogenerated electrons cause the degradation of MB. Change in absorbance of MB at 664 nm with time was measured under dark and irradiation, and, from the initial (*C_o_*) and the concentration at any time (*C*) of MB solution, the ratio of concentrations (*C/C_o_*) was determined (Figure 5). In order to establish the adsorption-desorption equilibrium of MB solution, MB solution was stirred in the dark for 30 min before the irradiation in all cases (Figure 5 inset).

Change of MB concentration with time suggested first-order reaction kinetics as reported in the literature [47] where MB concentration was related with the reaction time via the following equation 1;


(Equation-1) $$ln\frac{C}{C_{0}}=-kt$$

**Figure 6:**
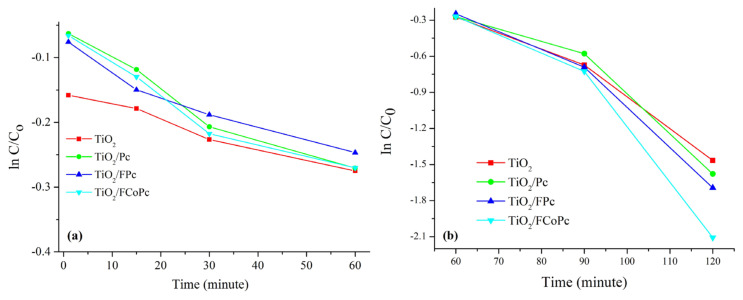
**a)** ln C/C_o_–time (region I), **b)** ln C/C_o_–time (region II) graphs for the photocatalytic degradation of MB for TiO_2_, TiO_2_/Pc, TiO_2_/FPc and TiO_2_/FCoPc nanofibers.

Here, *k* is a rate constant of the photodegradation of MB reaction, *t* is time, and *C* is the concentration of MB dye solution at a specific irradiation time. *k* values were obtained from the slope of the straight-lines of two regions (Region I= 0–60 minutes, Region II = 60–120 min) for the plot of *ln(C/C_0_)* versus *time* (Figure 6a, b).

According to the results given in Table 2, the k values in region I (k_I_) were almost 10 times lower than the k values in region II (k_II_), and TiO_2_/Pc and TiO_2_/FCoPc catalysts had the highest values in region I and region II, respectively. These results indicated that, although the interaction rate of TiO_2_/Pc was faster than the others, TiO_2_/FPc and TiO_2_/FCoPc seem more effective as the reaction progress (Figure 6a, b).

**Figure 7:**
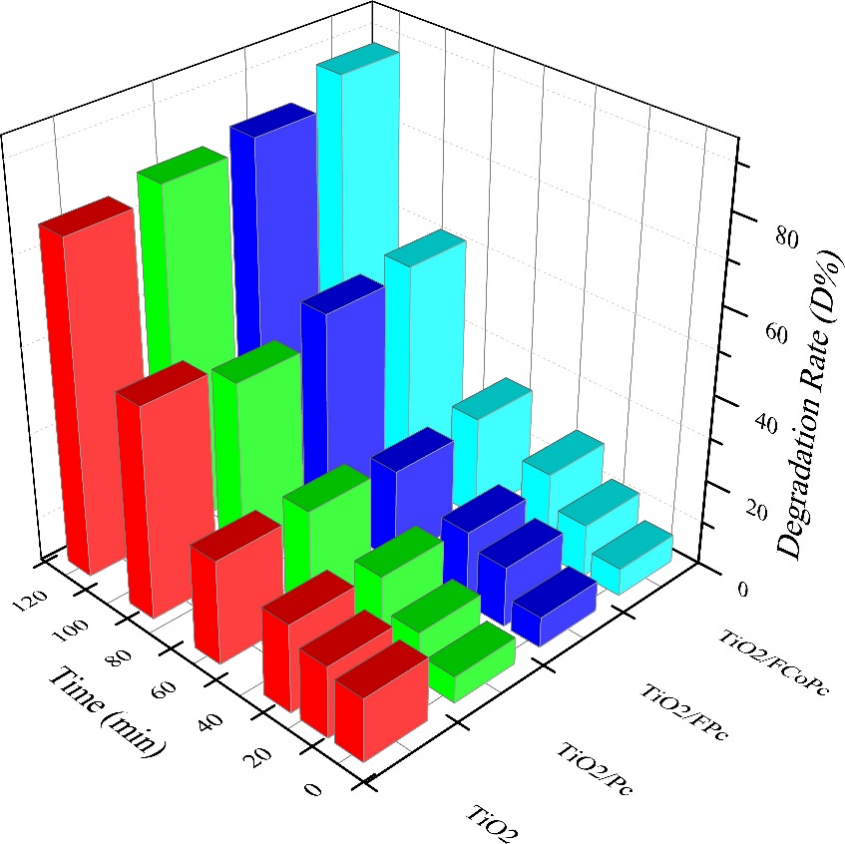
Photodegradation rate of MB for TiO_2_, TiO_2_/Pc, TiO_2_/FPc and TiO_2_/ FCoPc nanofibers.

The degradation rate (*D%*) was calculated with the following equation-2 (Figure 7):


(Equation-2) $$D\%=\frac{C_{0}-C}{C_{0}}\times 100\%$$

The photocatalytic reactivity order was found as TiO_2_/FCoPc > TiO_2_/FPc > TiO_2_/Pc > TiO_2_. The corresponding degradation rates of MB reached about 88% within 120 min for TiO_2_/FCoPc, which was in parallel with k values, due to the largest surface area of the TiO_2_/FCoPc nanofibers as can be seen from SEM images (Figure 3d).

**Figure 8:**
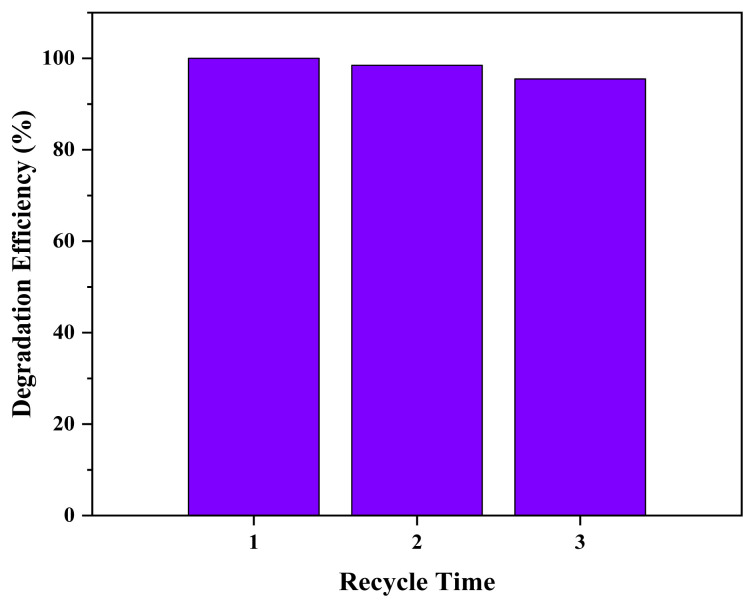
Recycling properties of the photocatalytic degradation of MB using TiO_2_/FCoPc as photocatalyst.

To determine the photocatalyst’s stability, which is critical for their practical applications, the TiO_2_/FCoPc was recovered by washing distilled water and used many times to degrade fresh MB solutions. Each time, the catalyst was dried in an oven at 50 °C, without any further modification. Figure 8 shows that, after reuse, the TiO_2_/FCoPc nanofiber retained high catalytic activity (100% to 98.5% and 95.51% by 1st, 2nd use, and 3rd use, respectively) with only approximately 6% loss in photocatalytic performance after the 3rd cycle.

According to these results, it can be suggested that the efficiency of TiO_2_/FCoPc as a photocatalyst in photocatalytic degradation reactions is similar or higher than the related studies in the literature [48–50] and can be suggested as a promising candidate for the removal of organic pollutants from wastewater.

**Figure 9:**
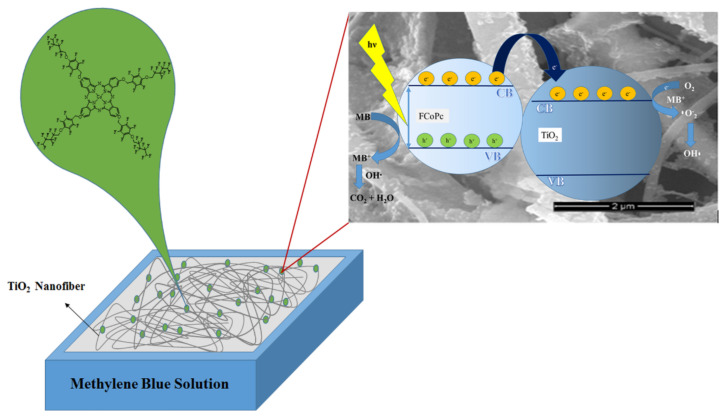
Schematic view of the degradation mechanism for MB by TiO_2_/FCoPc photocatalyst.

Based on the studies in the literature on photocatalytic degradation of organic pollutants in aqueous solutions [51–55], a possible mechanism for photocatalytic degradation of MB in UV light irradiation with FCoPc/TiO_2_ nanospun was schematically clarified in Figure 9, and the mechanism for photocatalytic degradation of MB was suggested as follows (Equations a–i):


(a) $$\text{MPc}+\text{h}v\to {\text{MPc}}^{*}$$

(b) MPc*+O2→MPc+O2(or O12)

(c) $${\text{MPc}}^{*}+{\text{TiO}}_{2}\to \text{MPc}{•}^{+}+{\text{TiO}}_{2}\;({\text{e}}^{-})$$

TiO_2_ nanofibers were first photoexcited to generate electron/hole (e^−^/h^+^) pairs. The electrons on the conduction band (CB) of TiO_2_ can be trapped to the O_2_ for generation of •O_2_^−^, which is the most necessary active material for photocatalytic degradation of MB. Photogenerated holes on the valence band (VB) of TiO_2_ were expected to react with H_2_O or OH^−^ to create •OH because the potential of occurrence was lower than the VB of TiO_2_.


(d) TiO2 (ecb-)+O2(or O12)→TiO2+O2•-

(e) $${\text{O}}_{2}{•}^{-}+{\text{H}}_{2}\text{O}\to {\text{HO}}_{2}•+{\text{OH}}^{-}$$

(f) $${\text{HO}}_{2}•+{\text{H}}_{2}\text{O}\to {\text{H}}_{2}{\text{O}}_{2}+\text{HO}•$$

(g) $${\text{H}}_{2}{\text{O}}_{2}\to 2\text{HO}•$$

(h) $$\text{HO}•+\text{MB}\to {\text{CO}}_{2}+{\text{H}}_{2}\text{O}$$

(i) $$\text{MPc}{•}^{+}+\text{MB}\to \text{MPc}+\text{MB}{•}^{+}$$

The TiO_2_ nanofibers serve as an electron trap for the activated surface adsorbed FCoPc dye. The trapped electron stimulates active oxygen species for later growth. Additionally, the active oxygen species, the by-produced radical cation FCoPc•^+^ has already interacted with MB and induces MB photodegradation. Because no valence band hole is created in the TiO_2_ nanofibrous, the nanofibrous FCoPc/TiO_2_ avoids recombination of the internal charge. TiO_2_ only acts as an electron mediator in this cycle, and the dye as a sensitizer.

## 4. Conclusion

TiO_2_ nanofiber was successfully fabricated from TBT precursor and firstly in-situ synthesis of Pc’s on electrospun TiO_2_ nanofiber was performed by the solvothermal process for photocatalytic degradation of MB. For this purpose, photocatalytic activity of a new type of fluor containing phthalocyanine (FPc) was comparatively investigated with non-flourinated Pc and fluor containing cobalt Pc (FCoPc). Here, the effect of cobalt metal ion and peripheral fluorinated groups on the design of the photocatalyst was studied. The structures and morphologies of TiO_2_/Pc nanofibers were characterized using XRD, SEM, EDX, and UV–Vis absorption spectra.

The kinetic studies of photocatalytic degradation showed that while the catalytic effects of photodegradation of the Pcs in the dark are less than that of TiO_2_, Pcs have absorption in the UV region, which increases light efficiency, and showed better catalytic effect under light. The TiO_2_/FCoPc nanofibers exhibited a higher catalytic activity of photodegradation for MB than the pure TiO_2_, TiO_2_/Pc, or TiO_2_/FPc nanofibers under UV-light irradiation after 90 min due to the advantage of fluorinated groups. The presence of cobalt improves this efficiency. According to recycling results, TiO_2_/FCoPc nanofiber was found to be suitable for multiple uses. Based on all results, TiO_2_/FCoPc photocatalyst may be a promising candidate for the purification of organic pollutants from wastewater.

## Figures and Tables

**Table 1:** EDX results of **(a)** TiO_2_, **(b)** TiO_2_/Pc, **(c)** TiO_2_/FPc and **(d)** TiO_2_/FCoPc nanofibers.

Element	Wt%			
	(a)	(b)	(c)	(d)
**Ti** **O** **N** **F** **Co**	50.29 49.70	50.35 42.27 7.37	49.57 34.57 5.37 10.48	48.06 35.46 4.38 11.51 0.58
**Total**	100.00	100.00	100.00	100.00

**Table 2:** Effect of different type of photocatalysts on the rate constant *k* and the total removal of photocatalytic MB degradation.

Sample name	k (min^−1^)		Total removal (%)
	Region I	Region II	
**TiO** **_2_**	0.0020	0.0199	77
**TiO** **_2_** **/Pc**	0.0036	0.0218	80
**TiO** **_2_** **/FPc**	0.0028	0.0240	82
**TiO** **_2_** **/FCoPc**	0.0031	0.0310	88
